# Breeding Guild Determines Frog Distributions in Response to Edge Effects and Habitat Conversion in the Brazil’s Atlantic Forest

**DOI:** 10.1371/journal.pone.0156781

**Published:** 2016-06-07

**Authors:** Rodrigo B. Ferreira, Karen H. Beard, Martha L. Crump

**Affiliations:** 1 Department of Wildland Resources and the Ecology Center, Utah State University, Logan, Utah, United States of America; 2 Laboratório de Ecologia de Populações e Conservação, Universidade Vila Velha, Vila Velha, ES, Brazil; 3 Department of Biology and the Ecology Center, Utah State University, Logan, Utah, United States of America; University of Sydney, AUSTRALIA

## Abstract

Understanding the response of species with differing life-history traits to habitat edges and habitat conversion helps predict their likelihood of persistence across changing landscape. In Brazil’s Atlantic Forest, we evaluated frog richness and abundance by breeding guild at four distances from the edge of a reserve: i) 200 m inside the forest, ii) 50 m inside the forest, iii) at the forest edge, and iv) 50 m inside three different converted habitats (coffee plantation, non-native *Eucalyptus* plantation, and abandoned pastures, hereafter matrix types). By sampling a dry and a wet season, we recorded 622 individual frogs representing 29 species, of which three were undescribed. Breeding guild (i.e. bromeliad, leaf-litter, and water-body breeders) was the most important variable explaining frog distributions in relation to edge effects and matrix types. Leaf-litter and bromeliad breeders decreased in richness and abundance from the forest interior toward the matrix habitats. Water-body breeders increased in richness toward the matrix and remained relatively stable in abundance across distances. Number of large trees (i.e. DBH > 15 cm) and bromeliads best explained frog richness and abundance across distances. Twenty species found in the interior of the forest were not found in any matrix habitat. Richness and abundance across breeding guilds were higher in the rainy season but frog distributions were similar across the four distances in the two seasons. Across matrix types, leaf-litter species primarily used *Eucalyptus* plantations, whereas water-body species primarily used coffee plantations. Bromeliad breeders were not found inside any matrix habitat. Our study highlights the importance of primary forest for bromeliad and leaf-litter breeders. We propose that water-body breeders use edge and matrix habitats to reach breeding habitats along the valleys. Including life-history characteristics, such as breeding guild, can improve predictions of frog distributions in response to edge effect and matrix types, and can guide more effective management and conservation actions.

## Introduction

Rapid habitat loss in the tropics has increased our need to understand how species respond to novel landscape features, such as edges and human-modified habitats [1,2,3,4]. Due to their great conservation implications, edge effects are one of the most studied topics in landscape ecology; however, because they influence a large number of variables, their role in species occurrences is complex and depends greatly upon the species studied. Examples of the complexities involved in studying edge effects include the wide range of distances that different edge effects can penetrate the forest [5]; the ability of edge effects to change over time with seasonal variation [6,7]; and the idea that different surrounding converted habitats (hereafter matrix types) may influence edge effects differently [8,9,10].

The degree of structural similarity between the forest interior and a matrix habitat may be the most important factor influencing species responses to edge and matrix habitats [11,12]. However, few studies carried out in tropical forests have evaluated the ability of different matrix types to influence edge effects and to harbor different species [3,9,13]. One hypothesis is that forest-associated species will use a matrix habitat that has low structural contrast with forests more than a matrix habitat with high structural contrast with forests [9]. For example, mature stands of *Eucalyptus* adjacent to primary forest are reported to have greater faunal richness than other agricultural matrices [14]. This type of information is essential to rank the conservation value of each matrix type according to its influence on species persistence [3]. Amphibians might be particularly sensitive to edge effects and matrix habitats because expected changes in temperature, humidity, wind speed, and soil moisture might increase their susceptibility to desiccation [15]. In addition, because frogs use a variety of reproductive habitats, including ponds, streams, bromeliads, and leaf litter, their response to habitat changes is expected to vary across breeding guilds [16,17].

More specifically, studies conducted in the highly disturbed Brazil’s Atlantic Forest (14.5% of the area is currently forest remnants) [18] show that certain reproductive modes of amphibians are more vulnerable to landscape alterations than others [1,2,19,20]. For example, because water-body breeders have different life history stages that use different habitats, separation of these habitats (termed “habitat split”) greatly affects them due to the risk associated with migrating from upland forest to reproductive habitats in the valleys [19,20]. Furthermore, bromeliad breeders may not often occur in matrix habitats or small forest fragments because bromeliads are often absent in these habitats [1]. In a lowland region of Brazil’s Atlantic Forest, Pardini et al. [21] found that forest-specialist leaf-litter breeders tended to avoid edges in large fragments, while Dixo & Martins [22] found no difference in the richness and abundance of leaf-litter breeders between edges and the interior of large fragments. Dixo & Martins [22] suggested that the lack of a detectable edge effect on leaf litter breeders may be due to the different types of matrix habitats surrounding the forest fragments in their study.

Although most land area across the Atlantic Forest has been converted to other land uses, the use of these different matrix types by frog species and their influence on edge effects remains largely unknown. Furthermore, much of the frog diversity of the Atlantic Forest is still being discovered and little is known concerning frog responses to landscape alteration as compared to studies conducted across temperate regions. The objective of this study was to investigate how frog richness and abundance, particularly across different breeding guilds, change with distances from the forest edge and with the three dominant matrix types in a mountainous region of Atlantic Forest. To help understand the mechanisms driving frog responses to these landscape changes, we also investigated how frog richness and abundance are related to habitat characteristics and microclimatic variables.

## Materials and Methods

### Study region

**Fig 1:**
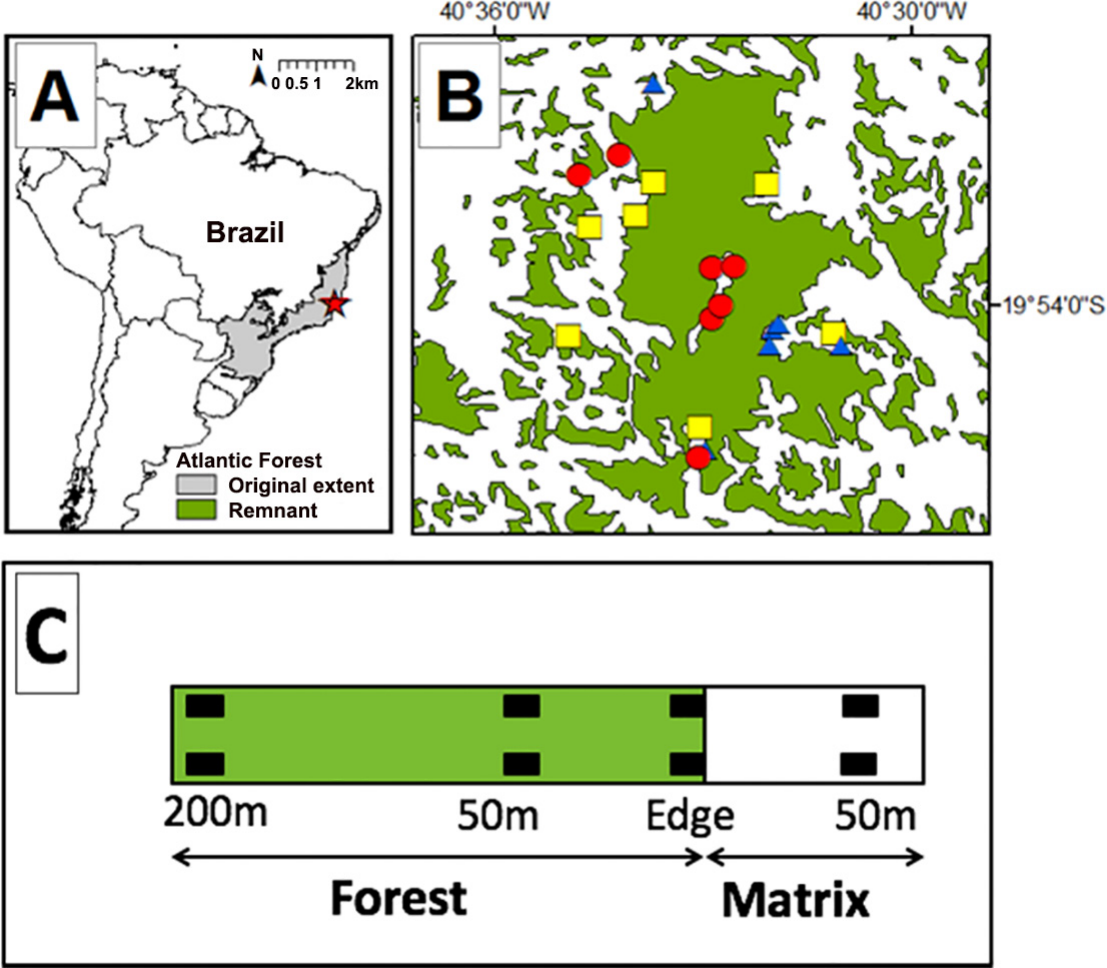
Study sites and sampling design. (A) Location of Santa Teresa municipality in the original extent of Brazil’s Atlantic Forest, (B) 21 sampled sites, adjacent to abandoned pasture (red circle), coffee plantation (blue triangle), and *Eucalyptus* plantation (yellow square), located within and around a biological reserve, and (C) sampling design showing a 250 m transect with paired plots (black square) by distance from the forest edge.

Research was conducted within and around the Reserva Biológica Augusto Ruschi (hereafter REBIO; Fig 1A; 19°45’- 20°00’ S, 40°27’- 40°38 W; 3,598 ha), in Santa Teresa, Espírito Santo state, Brazil. REBIO is in the northern portion of the Serra do Mar ecoregion in the Atlantic Forest biome and is classified as montane and sub-montane rain forest composed of moist broadleaf trees [23,24]. Santa Teresa was forested until the arrival of European settlers in 1874. Today this municipality has 42% forest cover [18]. The landscape of Santa Teresa is typical of mountainous regions in this biome; forest remnants are mostly restricted to hilltops, and water bodies (i.e. pond, stream, etc.) are located in the valleys, which are dominated by different types of human-modified matrix (e.g., coffee plantations, *Eucalyptus* spp. plantations, abandoned pastures, and settlements).

Santa Teresa’s climate is classified as humid-subtropical (Cwa-Cfa) according to Köppen-Geiger’s classification [25]. The dry season is mostly from May to August and the rainy season is from September to April. Mean annual precipitation is 1868 mm with highest rainfall in November and lowest in June, when the mean rainfall is less than 60 mm [26]. Mean annual temperature is 20°C, with minimum and maximum monthly temperatures averaging 14.3 C and 26.2 C, respectively [27].

### Sampling design

We surveyed amphibians at 21 sites (elevational range = 793–908 m) within and around the REBIO (Fig 1B). Each site was surveyed once from September to December 2012 (rainy season) and once from June to July 2013 (dry season). Sites comprised seven replicates of each of the three most widespread matrix types in this region (sun-grown coffee plantation, non-native *Eucalyptus* spp. plantation, and abandoned pastures).

At each site, we established a 250-m transect that ran perpendicular to the forest edge, from 50 m inside the matrix to 200 m inside the forest reserve. Along each transect, we surveyed at four distances: i) 50 m inside the matrix, ii) at the forest edge, iii) 50 m inside the forest, and iv) 200 m inside the forest (Fig 1C). At each distance on each transect, we established two 5 m x 5 m plots (hereafter paired-plots) 5 m from each other for measuring frog richness and abundance, microclimate variables, and habitat characteristics.

Sites were chosen in and around the REBIO to minimize potential confounding factors, such as fragment area and degree of isolation [28]. We selected sites that met the following criteria: i) matrix area was at least 100 m x 100 m; ii) *Eucalyptus* plantations were between four and seven years old; iii) coffee plantations were all sun-grown (i.e., no shade trees) and at a mature stage (i.e., harvesting stage); and iv) abandoned pastures were between 10 and 20 years old. We avoided selecting sites with human disturbance inside the forest during the last 10 years (e.g., bromeliad harvesting, heavy logging, and cattle).

### Frog sampling

This study was carried out in strict accordance with the recommendations in the Guidelines for euthanasia of animals from the veterinary medical association of both Brazil and United States of America [29,30]. Research protocol was approved by Instituto Chico Mendes de Conservação da Biodiversidade (ICMBio, Permit Number: 28607–3) and Institutional Animal Care and Use Committee of Utah State University (IACUC-USU, Permit Number 2002).

We hand-captured frogs in the leaf litter, in bromeliads and on the vegetation up to 2 m off the forest floor during nocturnal surveys from 1800 to 2300 hr. Four people worked simultaneously by moving the leaf litter for 20 minutes to survey each 5 m x 5 m plot.

We placed captured frogs in moist plastic tubes or plastic bags to prevent dehydration, and later brought them to the laboratory for identification. We released most frogs at the same site the following day; some frogs were euthanized because the amphibian taxonomy of the region is incomplete. We euthanized the frogs by ventral application of 7.5% to 10% benzocaine, fixed them in 10% formalin, and preserved them in 70% ethanol within one to five days of fixation. These specimens were deposited in the collections of Museu Nacional, Universidade Federal do Rio de Janeiro (MNRJ), State of Rio de Janeiro and the Museu de Biologia Mello Leitão (MBML), State of Espírito Santo, both in Brazil.

### Species traits

We classified each species according to its breeding habitat [bromeliad guild (lays eggs in bromeliads), leaf-litter guild (lays eggs on the forest floor), rock guild, or water-body guild (lays eggs in pond, river, or stream)]. We based classifications on Haddad et al. [31] and our field and laboratory observations along with expert observation, as necessary.

### Environmental variables

To measure microclimate variables, we placed a data logger (Onset HOBO U12-012) in each paired-plot to measure air temperature, air relative humidity, and light intensity during the 24 hours prior to frog sampling. We used a digital thermometer pistol to measure leaf-litter temperature from two corners of each plot. We used a portable weather station (Kestrel 2500) to measure wind speed from each paired-plot.

To measure habitat characteristics in each plot, we counted all trees and characterized them according to diameter at breast height (DBH) as: i) large trees (DBH > 15 cm), ii) medium-sized trees (DBH between 5 and 15 cm), and iii) small trees (DBH <5 cm). We counted tank bromeliads (Bromeliaceae) within 2 m height off the forest floor. We measured the leaf-litter depth in the four corners of each plot. We used a spherical densitometer to estimate the percent canopy cover in each plot.

### Statistical analysis

We employed generalized linear mixed models (GLMMs) to evaluate how frog richness (number of species) and abundance (total number of individuals) change with distance from edge (hereafter ‘Distance’), breeding guild (hereafter ‘Guild’), season, matrix type (hereafter ‘Matrix’), and environmental variables (microclimate variables and habitat characteristics). First, we tested 15 models using the full dataset to evaluate the main effects of ‘Distance’, ‘Guild’, ‘Matrix’, ‘Season’ and all possible interactions. Second, we tested eight models that included the frogs collected at each distance separately, except in the matrix because of the low number of collected frogs, to evaluate the effect of ‘Guild’, ‘Matrix’, ‘Season’ and all possible interactions. Finally, we tested another 10 models by taking the best-fitting model from our overall analysis and including each environmental variable as an interaction term to evaluate if any of these variables improved model fit.

For each predictive model, we assessed the effects of the fixed factors using a mixed model with two random effects factors: site within matrix type and distance within site. We specified a Poisson distribution with a log link. These analyses were conducted using the package *lme4* [32]. Because we studied a mountainous region, ‘elevation’ was included as “offset” in the models to address differences in elevation both across distances within the same transect and across sites.

Models were compared using an information theoretic approach, with lower values of Akaike’s information criterion corrected for small sample size (AIC_c_) indicating better-fitting models [33]. We also calculated ΔAIC_c_ (difference in AIC_c_ for each model from the most parsimonious model) and wAIC_c_ (AIC_c_ weight). Visual inspection of residual plots did not reveal any obvious deviations from homoscedasticity or normality.

We found no difference in habitat variables between the paired 5 m x 5 m plots sampled in the same transect, distance from edge, and season (Wilcoxon signed-rank test; package *stats*). Consequently, we summed some variables (tree structure and number of bromeliads) and took the mean of others (leaf-litter depth and canopy cover) from these paired-plots for analysis. We also summed frog richness and abundance found in these paired-plots.

Prior to analysis, we used variance inflation factors (VIF) to assess collinearity among air temperature, relative humidity, light intensity, and leaf-litter temperature using the package *vegan* [34]. We also visually inspected scatterplots using the package *corrgram* [35]. Leaf-litter temperature was excluded because it was correlated with air temperature. Mean, maximum, and minimum measurements of the other microclimate variables were highly correlated and were excluded from the analysis. Instead, we used the range (difference between maximum and minimum) for air temperature (hereafter ‘temperature range’), relative humidity (hereafter ‘humidity range’), and light intensity (hereafter ‘light range’) because VIF was smaller than 3. Resulting environmental variables were standardized to a mean of zero and a standard deviation of one to improve convergence of the fitting algorithm and to place the estimated coefficients on the same scale [36].

Due to small sample sizes inside the matrix, we used a Pearson’s chi-square exact test (χ2) to investigate whether richness and abundance of each breeding guild differed across ‘matrix type’. We also used Pearson’s chi-square exact test to evaluate the difference of richness and abundance between seasons across distances and breeding guilds. We conducted these chi-square tests using a Monte Carlo simulation based on 999 replicates with the package *MASS* [37]. We performed one-way analysis of variance to test for differences of environmental variables across both edges and matrix types. We used package *agricolae* [38] to run Tukey’s Honestly Significant Difference method (Tukey HSD) to control Type I error among pairwise mean comparisons. All analyses were conducted in version 3.0.3 of R software [39].

## Results

We recorded 622 individual frogs representing nine families and 29 species across the 168 paired-plots (4 distances x 21 sites x 2 seasons) (S1 Table). We documented a mean of 3.7 (± 4.0 standard error) individuals and 2.3 (± 2.1 standard error) species per paired-plot. We found three undescribed species: *Brachycephalus* sp., *Ischnocnema* sp. (aff. *parva*) 1, and *Ischnocnema* sp. (aff. *parva*) 2. We recorded 562 individuals of 12 leaf-litter breeding species, 37 individuals of 12 water-body breeding species, 22 individuals of four bromeliad breeding species, and one individual rock breeding species. We recorded 387 individuals of 27 species during the rainy season and 235 individuals of 17 species during the dry season.

‘Distance * Guild’ was the best-fitting model for frog richness (wAIC_c_ = 0.99) and abundance (wAIC_c_ = 0.99) across the landscape (Table 1). ‘Distance * Guild’ remained the best-fitting model for richness (wAIC_c_ = 0.73) and abundance (wAIC_c_ = 0.74) even after excluding the three most abundant species from the dataset (*A*. *glandulata*, *H*. *binotatus*, and *I*. cf. *parva* 1 represented 68% of total individuals) (S2 Table). Furthermore, ‘Guild’ was the best-fitting model for richness and abundance by analyzing each distance inside the forest separately (S3 Table). Richness and abundance were higher in the rainy season regardless of ‘Distance’ (S1 Fig) or ‘Guild’ (S2 Fig). No environmental variable improved model fit for frog richness or abundance (Table 2). Within these environmental models, however, ‘Distance * Guild * Large trees’ was the best-fitting model for richness (wAIC_c_ = 0.97) and ‘Distance * Guild * Total bromeliads’ was the best model for abundance (wAIC_c_ = 0.89) (Table 2).

**Table 1:** Model comparison of frog richness and abundance. Response variables evaluated in relation to 'Breeding guild', 'Distance', 'Matrix type', and 'Season' across 21 sites in the mountainous region of Brazil’s Atlantic Forest.

Models	Richness			Abundance		
	AIC_c_	ΔAIC_c_	wAIC_c_	AIC_c_	ΔAIC_c_	wAIC_c_
Distance * Guild	570.99	0	0.99	665.19	0	0.99
Distance * Guild * Season	585.47	14.48	0	674.31	9.124	0
Null	690.84	119.84	0	891.76	226.57	0
Distance	679.72	108.73	0	872.84	207.65	0
Guild	590.75	19.76	0	699.53	34.34	0
Matrix	694.84	123.84	0	895.80	230.61	0
Season	673.71	102.72	0	856.89	191.70	0
Guild * Matrix	599.38	28.39	0	707.62	42.43	0
Guild * Season	589.18	18.19	0	691.74	26.55	0
Distance * Guild * Matrix	599.39	28.40	0	680.73	15.54	0
Guild * Matrix * Season	611.77	40.78	0	714.25	49.06	0
Distance * Matrix	682.56	111.57	0	877.76	212.57	0
Distance * Season	668.90	97.91	0	843.911	178.72	0
Matrix * Season	681.74	110.75	0	862.94	197.74	0
Distance * Guild * Matrix * Season	697.38	126.39	0		771.989	106.79	0

**Table 2:** Model comparison of frog richness and abundance. Response variables evaluated in relation to environmental variables (microclimate and habitat characteristics).

Models	Richness				Abundance		
	AIC_c_	ΔAIC_c_	wAIC_c_		AIC_c_	ΔAIC_c_	wAIC_c_
**Reference model**							
Distance * Guild	570.99	0.00	1.00		665.19	0.00	0.99
Ref. model*Env. var.							
Large trees	579.24	0.00	0.97	Bromeliads	667.43	0.00	0.89
Light range	587.14	7.90	0.02	Wind	669.64	2.21	0.11
Leaf-litter depth	588.34	9.10	0.01	Large trees	672.95	5.52	0.03
Bromeliads	593.17	13.93	0.00	Small trees	674.19	6.76	0.02
Small trees	594.19	14.95	0.00	Canopy cover	676.21	8.78	0.01
Temp. range	601.01	21.77	0.00	Leaf-litter depth	676.99	9.56	0.00
Humidity range	597.81	18.57	0.00	Temp. range	678.90	11.47	0.00
Medium-sized trees	600.39	21.15	0.00	Humidity range	679.22	11.79	0.00
Canopy cover	595.54	16.30	0.00	Light range	684.11	16.69	0.00
Wind	599.10	19.86	0.00	Medium-sized trees	693.51	26.09	0.00

**Fig 2:**
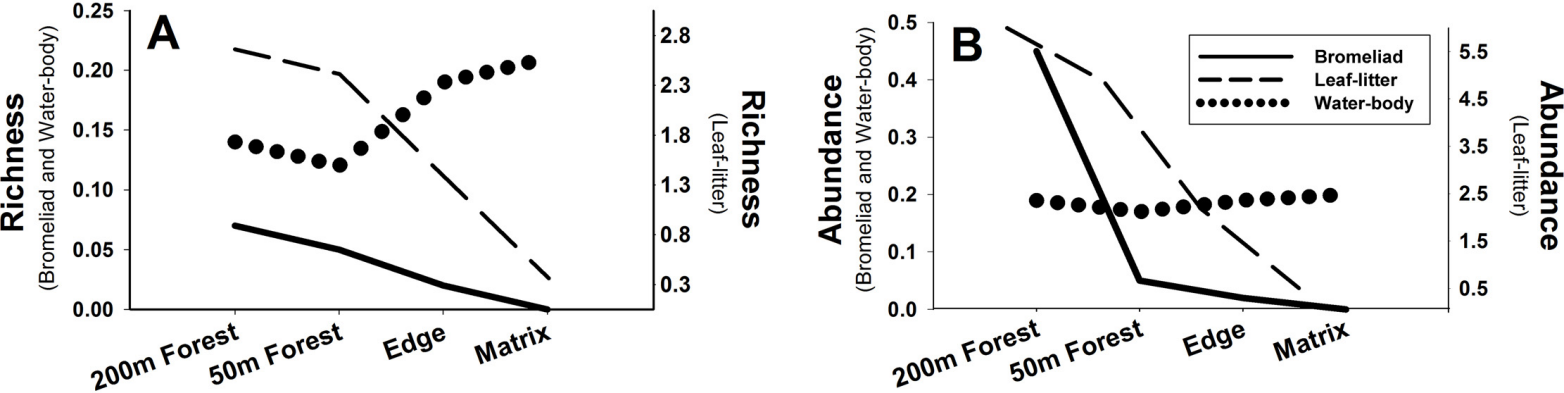
Response to edge effect by breeding guild. (A) Mean richness and (B) abundance of breeding guilds across distance from the forest edge across 21 sites.

Leaf-litter breeders had higher richness and abundance at every distance as compared to bromeliad and water-body breeders in both dry and rainy seasons (Fig 2; S1 Table). Bromeliad and leaf-litter breeders decreased in richness and abundance from the forest interior toward the matrix (Fig 2). Water-body breeders increased in richness toward the matrix and remained relatively stable in abundance across distances (Fig 2). The number of large trees and bromeliads increased toward forest interior, whereas the range of microclimate variables tended to decrease toward forest interior (Table 3).

**Table 3:** Microclimate variables and habitat characteristics. Measurements conducted by 'Distance' across 21 sites. Values are mean ± standard deviation.

Variables (units)	Matrix	Edge	50m Forest	200m Forest
**Microclimate variables**				
Temp. average (°C)	18.2 ± 1.9	17.4 ± 1.8	16.9 ± 1.7	17.1 ± 1.7
Temp. range (°C)	11.8 ± 4.1	7.2 ± 2.7	5.3 ± 1.9	5.5 ± 2.2
Humidity average (%)	91.6 ± 4.2	92.4 ± 8.9	96.8 ± 3.6	96.6 ± 3.8
Humidity range (%)	31.6 ± 13.9	18.8 ± 9.8	9.8 ± 9.5	10.6 ± 9.7
Light average (lx)	1459.8 ± 968.4	491.5 ± 484.5	119.9 ± 126.6	205.9 ± 330.4
Light range (lx)	13225.4 ± 8091.1	6292.6 ± 5663.6	3071.2 ± 3685.5	2837.5 ± 3384.6
Wind speed (Km/h)	2.4 ± 3.85	1.85 ± 3.6	1.7 ± 3.7	1.5 ± 1.4
**Habitat characteristics**				
Number of bromeliads	0.02 ± 0.1	1.6 ± 2.3	4.5 ± 5.6	7.4 ± 5.9
Small trees	20.2 ± 9.4	47.8 ± 14.0	42.7 ± 10.2	38.7 ± 8.4
Medium-sized trees	4.1 ± 2.6	10.9 ± 3.5	10.1 ± 2.1	10.1 ± 3.2
Large trees	1.2 ± 1.0	3.3 ± 2.5	4.6 ± 1.6	4.7 ± 1.8
Canopy cover (%)	66.2 ± 25.4	85.4 ± 14.1	91.4 ± 3.3	88.2 ± 11.7
Leaf litter depth (cm)	7.9 ± 2.9	8.6 ± 2.4	10 ± 4.0	12.7 ± 4.2

Of the frogs collected in the matrix habitats, five were water-body breeders, three were leaf-litter breeders, and one was a rock breeder, totaling 31 individuals of nine species (S1 Table). Bromeliad breeders were not found inside any matrix habitat. Four species were exclusively found in the matrix of which three were water-body breeders (S1 Table). Eight and 14 species found in the 50 m and 200 m forest plots, respectively, were not found in any matrix habitat. Across breeding guilds, these species found only inside the forest represent nine leaf-litter, four water-body and three bromeliad breeders.

**Fig 3:**
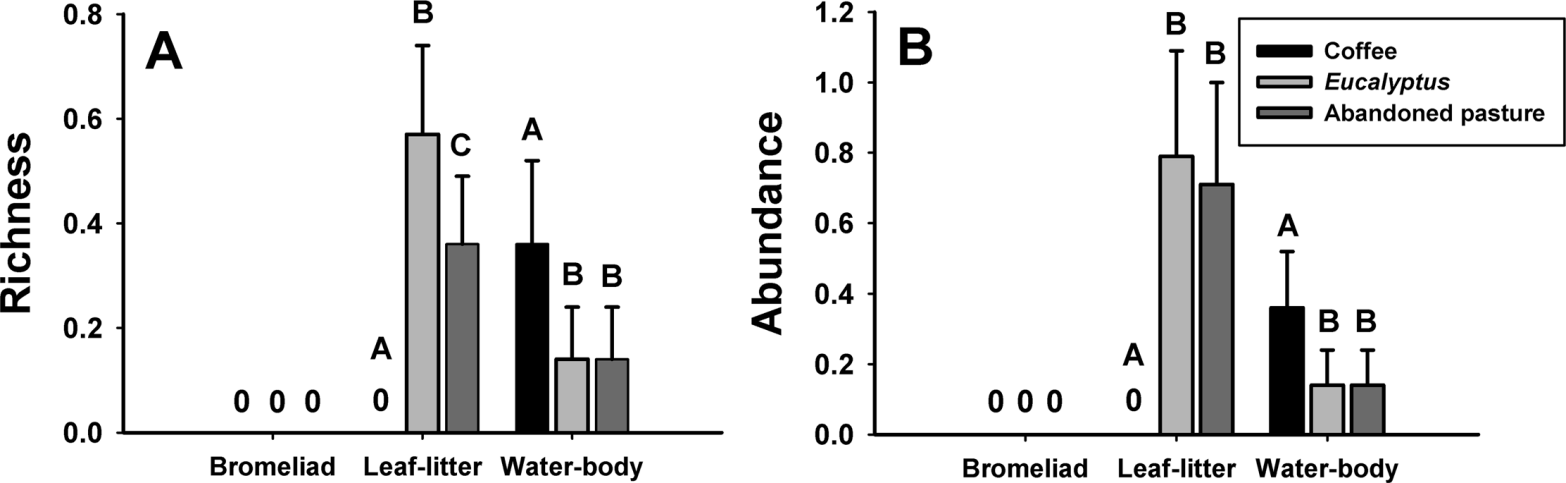
Use of matrix types by breeding guild. Mean and standard error of (A) richness and (B) abundance of breeding guilds inside seven replicates of each matrix type. Means with different letters are significantly different (χ^2^; P < 0.05).

Richness of leaf-litter breeders was higher in *Eucalyptus* than in abandoned pastures and coffee plantations, and abundance was higher in both *Eucalyptus* and abandoned pastures than in coffee plantations (Fig 3). Richness and abundance of water-body breeders were higher in coffee plantations than in the other matrix types (Fig 3). Leaf-litter breeders were not found in coffee plantations, whereas water-body breeders were found in all three matrix types (Fig 3). The only environmental variables that differed among the three matrix types were medium- and large-sized trees, which were higher in abandoned pastures and *Eucalyptus* plantations than in coffee plantations (Tukey HSD, P<0.05). There was no difference in any environmental variable at edges adjacent to the three matrix types (Tukey HSD, P>0.05).

## Discussion

The richness and abundance of frogs we studied in the mountainous region of Atlantic Forest varied across distances from forest edge (i.e., edge effect) and across matrix types (i.e., matrix effect). Breeding guild was the most important variable explaining these differences. More specifically, we found that bromeliad and leaf-litter species that do not require breeding habitats outside the forest had lower richness and abundance in edge and matrix habitats whereas water-body species that may require breeding habitats in the valleys increased in richness toward the matrix and remained relatively stable in abundance across distances. Richness and abundance across breeding guilds were higher in the rainy season but frog distributions were similar across the four distances in the two seasons. Across matrix types, leaf-litter species more often used *Eucalyptus* plantations, whereas water-body species more often used coffee plantations. Our data suggest that consideration of breeding habitat requirements can assist in predicting frog response to both edge effects and matrix habitats [1,16].

The increase in richness and abundance of bromeliad and leaf-litter breeders toward the forest interior may be in response to the increase of both large trees and bromeliads toward the forest interior. Pardini et al. [21] showed that forest-specialist leaf-litter breeders prefer the forest interior, which may be attributed to the higher concentration of large trees inside the forest. We observed a reduction in the range of microclimate variables (i.e. temperature, humidity and light intensity) toward the forest interior, which may be related to the increase in large trees. Trees buffer microclimate and also provide more leaf litter and suitable habitat for reproduction of species not dependent on bodies of water [7,40]. Furthermore, trees host epiphytic bromeliads, which may contribute to the observed increase of both bromeliads and bromeliad breeders toward the forest interior. As hypothesized, our results suggest that primary forest is more suitable for reproduction for bromeliad and leaf-litter breeders than matrix habitats.

We suggest that water-body breeders use edge and matrix habitats because they need to reach water bodies along the valleys [1,2,20]. Of the 12 water-body breeding species we observed, nine were forest specialists (as opposed to open habitat specialist or generalist; S1 Table), and forest specialists made up 84% of the individual water-body breeders collected. Based on this information, our data suggest that these individuals are likely just moving through these habitats.

Previous studies carried out in habitat fragments in the Atlantic Forest show that forest fragments disjunct from water bodies have lower richness and abundance of water-body breeders as compared to forests connected to these reproductive habitats [1,20]. Our study, however, was conducted in and around a reserve and resources required for water-body breeders are both inside the forest and in the matrix. The frogs in our study region appear to use water bodies in the valleys outside the reserve despite the risk of migration through a potentially inhospitable habitat. This might occur because of natal philopatry. It would be important to determine if this is the case and whether this movement is reducing their population densities. The fact that our study recorded only 16% of water-body breeding species ever recorded in Santa Teresa, compared to 57% of bromeliad breeders and 70% of leaf-litter breeders [41,42] indicates that most water-body breeders may be reproducing deeper than 200 m inside the forest reserve, and that perhaps those water-body breeders living near the edge of the reserve have already declined.

Contrary to other studies [6,7], edge effects were not influenced by seasonality. Richness and abundance of frogs were higher in the rainy season regardless of distance or guild. This result suggests that the response of frogs to edge effects may be studied in either season. Similar to other tropical regions, the rainy season is the reproductive season for most frogs at our study sites [2,43]. The dry season is less suitable for frog activity due to shorter photoperiod and lower temperature and humidity [44,45,46], and thus researchers are less likely to encounter frogs during the dry season.

Matrix type had no measurable effect on frog distributions or environmental variables in the forest edges or in the forest interior. This is surprising considering the lower abundance of medium- and large-sized trees inside coffee plantations compared to the other matrix types. On the other hand, the breeding guilds used the matrix types differently. Bromeliad and leaf-litter breeders were not found in coffee plantations possibly because coffee plantations in our study are open canopy. Studies have shown that shade-growth coffee plantations are suitable matrix type for frogs across Neotropical ecoregions [47]. Surprisingly, coffee plantation was the most used matrix type by water-body breeding species. This might be a result of location of water bodies and the history of the amphibians in the area rather than a preference for this matrix type. *Eucalyptus* plantation is the most forest-like matrix type in our studied region, which might explain the higher richness of leaf-litter breeding species in this habitat. Many studies have pointed out the importance of secondary forests for amphibians as compared to agricultural or plantation matrix types [21,48,49]. Abandoned pastures in our region are not becoming secondary forest, because secondary forest and other agricultural areas are being converted to *Eucalyptus* plantations. This landscape change could be detrimental to water-body breeders, considering that they had the lowest richness and abundance in *Eucalyptus* plantations.

## Conclusions

Our study tested for the importance of considering the influence of surrounding matrix type in edge effects on frogs; however, we found the interaction between matrix type and edge effects unimportant. For the first time, we showed that the edge effects for Atlantic Forest frogs were not influenced by different surrounding matrix types.

Our results agree with previous suggestions that primary forest is critical for the persistence of most frogs in Atlantic Forest [1,21,50]. The lower richness and abundance of bromeliad and leaf-litter frogs inside the matrix compared to the forest interior suggests that the conversion of the existing natural habitats to any type of matrix will have strong deleterious effects on these breeding guilds. However, the fact that three species were found exclusively in the matrix shows that these habitats are important for sustaining amphibian diversity.

The fact that water-body breeders are more associated with edge and matrix habitats in our study sites suggests that matrix quality could be important for these species as they migrate toward reproductive habitats in the valleys. Ferreira, Dantas & Tonini [2] showed that forest corridor connecting upland forests and water bodies in the valleys have higher richness and abundance of frogs than water bodies surrounded by *Eucalyptus* plantation and human construction. To conserve the diversity of breeding guilds of frogs in Atlantic Forest, we recommend that conservation initiatives focus on maintaining protected areas and improving the connection between upland forested areas and water bodies in the valleys.

## Supporting Information

S1 Fig Frog response to season across distance. Mean and standard error of (A) richness and (B) abundance of frogs across distance by season across 21 sites. Means with different letters are significantly different (χ^2^; P < 0.05).
(TIF)

S2 Fig Frog response to season by breeding guild. Mean and standard error of (A) richness and (B) abundance of frog’s breeding guilds by season across 21 sites. Means with different letters are significantly different (χ^2^; P < 0.05).
(TIF)

S1 Table List of the 29 species recorded in Santa Teresa municipality, southeastern Brazil. The list includes species traits and abundance by distance from the forest edge. Breeding guild: BR = bromeliad, LL = leaf litter, RW = rock wall, and WB = water body (pond, stream, or river). Forest association: F = forest dependent, O = open-habitat, and G = habitat-generalist. * disregarded in the statistical analysis.
(DOCX)

S2 Table Model comparison of frog richness and abundance. Response variables evaluated in relation to ‘Breeding guild’, ‘Distance’, and ‘Season’, after excluding the three most abundant frog species from the dataset.
(DOCX)

S3 Table Model comparison of frog richness and abundance. Response variables evaluated in relation to ‘Breeding guild’, ‘Matrix type’, and ‘Season’ for data collected in each of the three distances inside the forest separately.
(DOCX)
