# Supplementary figures and images for: Breeding Guild Determines Frog Distributions in Response to Edge Effects and Habitat Conversion in the Brazil’s Atlantic Forest
Source: PLoS One. 2016 Jun 7;11(6):e0156781. doi: 10.1371/journal.pone.0156781 (PMC4896733; doi:10.1371/journal.pone.0156781)

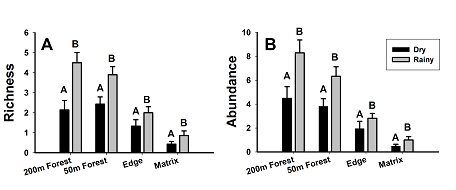

Supplement: S1 Fig — Mean and standard error of (A) richness and (B) abundance of frogs across distance by season across 21 sites. Means with different letters are significantly different (χ2; P < 0.05). (TIF) [file pone.0156781.s001.tif]

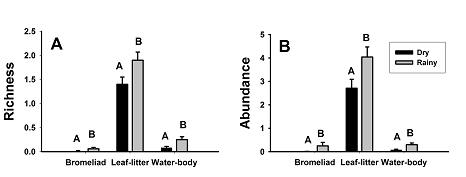

Supplement: S2 Fig — Mean and standard error of (A) richness and (B) abundance of frog’s breeding guilds by season across 21 sites. Means with different letters are significantly different (χ2; P < 0.05). (TIF) [file pone.0156781.s002.tif]
