# Supplementary material for: Breeding Guild Determines Frog Distributions in Response to Edge Effects and Habitat Conversion in the Brazil’s Atlantic Forest
Source: PLoS One. 2016 Jun 7;11(6):e0156781. doi: 10.1371/journal.pone.0156781 (PMC4896733; doi:10.1371/journal.pone.0156781)
Supplement: S1 Table — The list includes species traits and abundance by distance from the forest edge. Breeding guild: BR = bromeliad, LL = leaf litter, RW = rock wall, and WB = water body (pond, stream, or river). Forest association: F = forest dependent, O = open-habitat, and G = habitat-generalist. * disregarded in the statistical analysis. (DOCX) [file pone.0156781.s003.docx]

| **Frog species by family** | **Breeding guild** |  | **Abundance by distance** | | | | **Total**  **abundance** |
| --- | --- | --- | --- | --- | --- | --- | --- |
|  |  | **Forest association** | Matrix | Edge | 50m Forest | 200m Forest |  |
| **Brachycephalidae** |  |  |  |  |  |  |  |
| *Brachycephalus* sp. | LL | F | 0 | 0 | 1 | 0 | 1 |
| *Ischnocnema abdita* Canedo & Pimenta, 2010 | LL | F | 0 | 7 | 11 | 23 | 41 |
| *Ischnocnema* aff. *guentheri* Steindachner, 1864 | LL | F | 7 | 1 | 1 | 0 | 9 |
| *Ischnocnema nasuta* (Lutz, 1925) | BR | F | 0 | 0 | 1 | 0 | 1 |
| *Ischnocnema oea* (Heyer, 1984) | LL | F | 0 | 9 | 17 | 18 | 44 |
| *Ischnonema* (aff. *parva* ) 1 | LL | F | 0 | 11 | 48 | 76 | 135 |
| *Ischnonema* (aff. *parva* ) 2 | LL | F | 0 | 0 | 1 | 1 | 2 |
| *Ischnocnema verrucosa* Reinhardt and Lutken, 1862 | LL | F | 0 | 4 | 11 | 14 | 29 |
| *Ischnocnema* sp. | LL | F | 0 | 1 | 0 | 1 | 2 |
| **Craugastoridae** |  |  |  |  |  |  |  |
| *Euparkerella tridactyla* Izecksohn, 1988 | LL | F | 0 | 0 | 3 | 4 | 7 |
| *Haddadus binotatus* (Spix, 1824) | LL | F | 11 | 47 | 66 | 74 | 198 |
| **Cycloramphidae** |  |  |  |  |  |  |  |
| *Thoropa miliaris* (Spix, 1824) * | RW | G | 1 | 0 | 0 | 0 | 1 |
| *Zachaenus carvalhoi* Izecksohn, 1983 | LL | F | 0 | 0 | 1 | 2 | 3 |
| **Eleutherodactylidae** |  |  |  |  |  |  |  |
| *Adelophryne glandulata* Lourenço-de-Moraes et al., 2014 | LL | F | 3 | 10 | 44 | 34 | 91 |
| **Hylidae** |  |  |  |  |  |  |  |
| *Bokermannohyla caramaschii* (Napoli, 2005) | WB | F | 1 | 3 | 0 | 2 | 6 |
| *Hypsiboas faber* (Wied-Neuwied, 1821) | WB | G | 0 | 2 | 0 | 0 | 2 |
| *Hypsiboas semilineatus* (Spix, 1824) | WB | F | 2 | 0 | 0 | 0 | 2 |
| *Phasmahyla exilis* (Cruz, 1980) | WB | F | 0 | 0 | 0 | 2 | 2 |
| *Scinax alter* (Lutz, 1973) | WB | O | 2 | 0 | 0 | 0 | 2 |
| *Scinax arduous* Peixoto, 2002 | BR | F | 0 | 1 | 0 | 0 | 1 |
| *Scinax* sp. | WB | F | 0 | 0 | 1 | 1 | 2 |
| **Hylodidae** |  |  |  |  |  |  |  |
| *Crossodactylus* sp. | WB | F | 0 | 0 | 0 | 5 | 5 |
| **Leptodactylidae** |  |  |  |  |  |  |  |
| *Crossodactylodes bokermanni* Peixoto, 1983 | BR | F | 0 | 0 | 1 | 1 | 2 |
| *Crossodactylodes izecksohni* Peixoto, 1983 | BR | F | 0 | 0 | 0 | 18 | 18 |
| *Physalaemus crombiei* Heyer & Wolf, 1989 | WB | F | 0 | 2 | 0 | 0 | 2 |
| *Physalaemus cuvieri* Fitzinger, 1826 | WB | O | 2 | 0 | 0 | 0 | 2 |
| **Microhylidae** |  |  |  |  |  |  |  |
| *Chiasmocleis schubarti* Bokermann, 1952 | WB | F | 0 | 0 | 1 | 2 | 3 |
| **Odontophrynidae** |  |  |  |  |  |  |  |
| *Proceratophrys boiei* (Wied-Neuwied, 1824) | WB | F | 0 | 1 | 0 | 0 | 1 |
| *Proceratophrys paviotii* Cruz, Prado & Izecksohn, 2005 | WB | F | 2 | 1 | 4 | 1 | 8 |
| Total richness |  |  | 9 | 15 | 17 | 18 | - |
| Total abundance |  |  | 31 | 101 | 213 | 279 | 622 |
