# Supplementary material for: Breeding Guild Determines Frog Distributions in Response to Edge Effects and Habitat Conversion in the Brazil’s Atlantic Forest
Source: PLoS One. 2016 Jun 7;11(6):e0156781. doi: 10.1371/journal.pone.0156781 (PMC4896733; doi:10.1371/journal.pone.0156781)
Supplement: S2 Table — Response variables evaluated in relation to ‘Breeding guild’, ‘Distance’, and ‘Season’, after excluding the three most abundant frog species from the dataset. (DOCX) [file pone.0156781.s004.docx]

| **Models** | **Richness** | | |  | **Abundance** | | |
| --- | --- | --- | --- | --- | --- | --- | --- |
|  | AIC_c_ | ΔAIC_c_ | wAIC_c_ |  | AIC_c_ | ΔAIC_c_ | wAIC_c_ |
| Distance * Guild | **360.63** | **0** | **0.73** |  | **445.36** | **0** | **0.74** |
| Guild * Season | 364.07 | 3.43 | 0.13 |  | 447.76 | 2.39 | 0.23 |
| Guild | 364.09 | 3.46 | 0.13 |  | 452.71 | 7.34 | 0.01 |
| Guild * Matrix | 369.63 | 9.00 | 0 |  | 453.89 | 8.52 | 0 |
| Null | 413.72 | 53.09 | 0 |  | 456.23 | 10.86 | 0 |
| Distance | 418.28 | 57.65 | 0 |  | 457.68 | 12.31 | 0 |
| Matrix | 416.01 | 55.36 | 0 |  | 530.61 | 85.24 | 0 |
| Season | 406.39 | 45.75 | 0 |  | 535.36 | 89.99 | 0 |
| Distance * Guild * Matrix | 388.55 | 27.926 | 0 |  | 533.51 | 88.13 | 0 |
| Distance * Guild * Season | 376.87 | 16.24 | 0 |  | 519.49 | 74.13 | 0 |
| Guild * Matrix * Season | 382.64 | 22.01 | 0 |  | 467.92 | 22.55 | 0 |
| Distance * Matrix | 424.42 | 63.79 | 0 |  | 545.29 | 99.92 | 0 |
| Distance * Season | 415.88 | 55.25 | 0 |  | 528.13 | 82.76 | 0 |
| Matrix * Season | 408.02 | 47.39 | 0 |  | 502.82 | 57.45 | 0 |
| Distance * Guild * Matrix * Season | 599.41 | 238.77 | 0 |  | 680.74 | 235.37 | 0 |
