# Supplementary material for: Breeding Guild Determines Frog Distributions in Response to Edge Effects and Habitat Conversion in the Brazil’s Atlantic Forest
Source: PLoS One. 2016 Jun 7;11(6):e0156781. doi: 10.1371/journal.pone.0156781 (PMC4896733; doi:10.1371/journal.pone.0156781)
Supplement: S3 Table — Response variables evaluated in relation to ‘Breeding guild’, ‘Matrix type’, and ‘Season’ for data collected in each of the three distances inside the forest separately. (DOCX) [file pone.0156781.s005.docx]

| **Models by distance** | **Richness** | | |  | **Abundance** | | |
| --- | --- | --- | --- | --- | --- | --- | --- |
|  | AIC_c_ | ΔAIC_c_ | wAIC_c_ |  | AIC_c_ | ΔAIC_c_ | wAIC_c_ |
| **Edge** |  |  |  |  |  |  |  |
| Guild | **145.88** | **0.00** | **0.98** |  | **167.19** | **0.00** | **0.96** |
| Guild * Season | 154.25 | 8.37 | 0.02 |  | 173.59 | 6.41 | 0.04 |
| Guild * Matrix | 159.49 | 13.61 | 0.00 |  | 177.92 | 10.74 | 0.00 |
| Matrix | 170.85 | 24.97 | 0.00 |  | 202.64 | 35.46 | 0.00 |
| Season | 165.84 | 19.96 | 0.00 |  | 197.16 | 29.97 | 0.00 |
| Null | 166.33 | 20.45 | 0.00 |  | 198.09 | 30.91 | 0.00 |
| Season * Matrix | 174.72 | 28.84 | 0.00 |  | 203.46 | 36.27 | 0.00 |
| Guild * Season * Matrix | 194.06 | 48.18 | 0.00 |  | 210.32 | 43.13 | 0.00 |
| **50 m forest** |  |  |  |  |  |  |  |
| Guild | **179.02** | **0.00** | **0.93** |  | **215.07** | **0.00** | **0.85** |
| Guild * Season | 184.27 | 5.25 | 0.07 |  | 217.47 | 2.40 | 0.15 |
| Matrix | 217.07 | 38.05 | 0.00 |  | 274.87 | 59.81 | 0.00 |
| Season | 207.69 | 28.67 | 0.00 |  | 259.37 | 44.31 | 0.00 |
| Null | 212.66 | 33.64 | 0.00 |  | 270.38 | 55.31 | 0.00 |
| Guild * Matrix | 194.99 | 15.97 | 0.00 |  | 227.05 | 11.98 | 0.00 |
| Season * Matrix | 212.23 | 33.21 | 0.00 |  | 266.09 | 51.02 | 0.00 |
| Guild * Season * Matrix | 216.10 | 37.08 | 0.00 |  | 250.10 | 35.04 | 0.00 |
| **200 m forest** |  |  |  |  |  |  |  |
| Guild | **190.38** | **0.00** | **0.95** |  | **232.07** | **0.00** | **0.85** |
| Guild * Season | 196.27 | 5.89 | 0.05 |  | 235.65 | 3.59 | 0.14 |
| Guild * Matrix | 202.59 | 12.20 | 0.00 |  | 240.51 | 8.44 | 0.01 |
| Matrix | 222.94 | 32.55 | 0.00 |  | 314.52 | 82.46 | 0.00 |
| Season | 219.38 | 29.00 | 0.00 |  | 299.25 | 67.18 | 0.00 |
| Null | 223.58 | 33.21 | 0.00 |  | 314.17 | 82.10 | 0.00 |
| Season * Matrix | 222.62 | 32.24 | 0.00 |  | 301.15 | 69.08 | 0.00 |
| Guild * Season * Matrix | 230.91 | 40.53 | 0.00 |  | 267.84 | 35.77 | 0.00 |
